# Mobile phone text messaging intervention to improve alertness and reduce sleepiness and fatigue during shiftwork among emergency medicine clinicians: study protocol for the SleepTrackTXT pilot randomized controlled trial

**DOI:** 10.1186/1745-6215-15-244

**Published:** 2014-06-21

**Authors:** Paul Daniel Patterson, Charity G Moore, Matthew D Weaver, Daniel J Buysse, Brian P Suffoletto, Clifton W Callaway, Donald M Yealy

**Affiliations:** 1 Department of Emergency Medicine, School of Medicine, University of Pittsburgh, 3600 Forbes Avenue, Iroquois Bldg, Suite 400A, Pittsburgh, PA, USA; 2 Division of General Internal Medicine, School of Medicine, Department of Biostatistics, Graduate School of Public Health, University of Pittsburgh, 200 Meyran Avenue, Suite 300, Pittsburgh, PA, USA; 3 Department of Psychiatry and Clinical and Translational Science, University of Pittsburgh School of Medicine, 3811 O’Hara Street, E-1127 Pittsburgh, PA, USA

**Keywords:** Shiftwork, Sleepiness, Fatigue, Alertness, Emergency medicine, Randomized controlled trial

## Abstract

**Background:**

Mental and physical fatigue while at work is common among emergency medical services (EMS) shift workers. Extended shifts (for example 24 hours) and excessive amounts of overtime work increase the likelihood of negative safety outcomes and pose a challenge for EMS fatigue-risk management. Text message-based interventions are a potentially high-impact, low-cost platform for sleep and fatigue assessment and distributing information to workers at risk of negative safety outcomes related to sleep behaviors and fatigue.

**Methods/Design:**

We will conduct a pilot randomized trial with a convenience sample of adult EMS workers recruited from across the United States using a single study website. Participants will be allocated to one of two possible arms for a 90-day study period. The intervention arm will involve text message assessments of sleepiness, fatigue, and difficulty with concentration at the beginning, during, and end of scheduled shifts. Intervention subjects reporting high levels of sleepiness or fatigue will receive one of four randomly selected intervention messages promoting behavior change during shiftwork. Control subjects will receive assessment only text messages. We aim to determine the performance characteristics of a text messaging tool for the delivery of a sleep and fatigue intervention. We seek to determine if a text messaging program with tailored intervention messages is effective at reducing perceived sleepiness and/or fatigue among emergency medicine clinician shift workers. Additional aims include testing whether a theory-based behavioral intervention, delivered by text message, changes ‘alertness behaviors’.

**Discussion:**

The SleepTrackTXT pilot trial could provide evidence of compliance and effectiveness that would support rapid widespread expansion in one of two forms: 1) a stand-alone program in the form of a tailored/individualized sleep monitoring and fatigue reduction support service for EMS workers; or 2) an add-on to a multi-component fatigue risk management program led and maintained by employers or by safety and risk management services.

**Trial Registration:**

Clinicaltrials.gov NCT02063737, Registered on 10 January 2014

## Background

Fatigue is ‘a subjective, unpleasant symptom, which incorporates total body feelings ranging from tiredness to exhaustion creating an unrelenting overall condition which interferes with individual’s ability to function to their normal capacity’ [1]. Fatigue can be linked to inadequate or disturbed sleep and is a common problem among workers in industries that provide 24-hour/day services, including healthcare [2]. There is compelling evidence linking sleep, fatigue, and shiftwork to negative safety outcomes [3]. One study determined that fatigue at the time of a workplace injury was eight times more likely among healthcare workers that worked shifts longer than 12 hours in duration [4]. Scheduled naps, exercise, modifying sleep habits, and use of caffeine ameliorate the effects of poor sleep, fatigue, and decreased alertness [5-13].

Unfortunately, many shift workers may not adopt such solutions and commonly sacrifice sleep health for economic reasons, family work-life reasons, or other factors [2,14]. For these workers, solutions need to be tailored to their shiftwork lifestyle, have an impact, and be timely. For employers of these workers, solutions should be economically viable, modifiable to changes in shift structure, and scalable for an entire workforce.

Emergency medical services (EMS) clinicians are a group of workers where extended shifts, inconsistent shift patterns, poor sleep, and fatigue are common [14-17]. There are an estimated 19,000 EMS organizations and 800,000 to 1 million licensed EMS workers in the United States today [18]. EMS workers are often the first point of clinical contact for the acutely ill and injured. Many EMS workers work for extended periods (for example 24 hours), often consecutively, and may accumulate many hours of overtime [14-17]. Half of EMS workers sleep less than six hours per sleep period and many report poor sleep quality [16]. One-third of EMS workers report excessive daytime sleepiness [19]. High workload combined with limited or poor sleep contributes to half of all EMS workers reporting excessive mental and physical fatigue while at work [15,16].

Survey data show convincing relationships between sleep, fatigue, and safety outcomes in EMS workers [16]. The odds of injury are 1.9 times greater among fatigued EMS workers than the non-fatigued; after controlling for confounding [16]. The odds of making an error or experiencing a patient-related adverse event are 2.2 times greater among fatigued EMS workers than the non-fatigued [16]. Fatigue and poor sleep increase the likelihood of risky behaviors. The odds of engaging in behaviors that compromise patient or worker safety (such as driving at high speeds in an ambulance when potentially not necessary) are 3.6 times greater among EMS workers that report fatigue [16]. Unfortunately, shift structure in EMS, a potential contributor to fatigued EMS workers, is not widely regulated and there is little to no incentive to recognize or admit to fatigue while on the job. Few EMS agencies have an evidence-based fatigue risk management program, and there is limited evidence of any best practices for lessening fatigue in the EMS industry.

As in other shift work populations, popular strategies to address poor sleep and fatigue among shift workers include scheduled naps, shift length modification, caffeine, and mental and physical exercise [5-13,20]. However, the effectiveness of any one or combination of these strategies in an EMS organization has not been widely studied. Boudreaux *et al.* evaluated the impact of eliminating 24 hour shifts in favor of 12 hour standard shifts in one medium-sized EMS agency [20]. Short-term impacts included lower perceived burnout and a more positive perception of work-life balance [20]. These findings returned to pre-intervention levels after 12 months [20]. One study of 10 EMS workers in Japan showed that scheduled naps while on duty had no impact on reaction time or self-reported fatigue compared to workers without naps [21]. Wide variation in EMS worker shift schedules, sleep habits and behaviors, and knowledge of sleep hygiene and signs/symptoms of fatigue are obstacles for any intervention and may have impacted the findings of previous research. Other obstacles include employers preoccupied with day-to-day challenges that do not involve worker sleep or fatigue. Some organizations may unintentionally encourage extended shifts in order to maintain full staffing and reduce operating costs without knowledge of the impact on worker sleep, fatigue, and safety. Poor workplace safety culture is prevalent in many EMS organizations and may further impact the likelihood that employers invest in worker sleep hygiene and fatigue-reduction programs [22,23]. The complex nature of sleep and fatigue in EMS shift workers, combined with economic and systems-level operation pressures such as maintaining 24 hour readiness, demands that innovative solutions and strategies be tested and developed in an EMS setting.

There is growing enthusiasm and preliminary data to support the use of mobile technologies and real-time interventions using cell phones or smartphones [24,25]. More than 90% of Americans own a cell phone and 55% own a smartphone [26,27]. More than 80% of cell phone owners send or receive text messages, 60% access the internet on their phone, and 52% send or receive email via their cell phone or smartphone [26]. A 2012 systematic review of mobile health (mHealth) intervention studies identified a positive impact on select behaviors using text messaging of intervention materials and messages (smoking, diabetes control, weight loss, and asthma self-management) [25]. Other reviews identify many studies with positive findings, yet temper support for mHealth-based interventions without additional high quality randomized controlled trials [24].

Tailored mHealth interventions may benefit EMS worker sleep habits and behaviors related to fatigue. For example, adherence to a well-organized behavioral change program for sleep has been shown to reduce the signs and symptoms of insomnia and improve self-reported sleep health [28]. A common challenge with any behavioral intervention has been longterm adherence and maintenance [29,30]. Adherence may be impacted by an acute change in lifestyle (such as shiftwork patterning) or a change in motivation related to the loss of continued support, coaching, or ongoing engagement [29-31]. Prior studies show ongoing support and engagement via telephone or web-based contact can improve adherence to an intervention; yet results from strictly web-based interventions are generally less positive [32]. Preliminary results from behavioral change studies using text messaging show positive sustained adherence of behavioral change [33]. Text message-based interventions are a potentially high-impact, low-cost platform for promoting positive sleep habits, tracking sleepiness and fatigue, and distributing information to EMS shift workers at risk of negative safety outcomes related to sleep behaviors and fatigue.

In this paper, we present the study protocol for a controlled 90-day pilot trial with an assessment-only control group. This study has two objectives. First, we aim to determine the performance characteristics of a text messaging tool as the prime vehicle for delivery of a sleep and fatigue intervention for EMS shift workers. Second, we aim to determine if a short message service (SMS) program with tailored intervention messages is effective at reducing perceived sleepiness and/or fatigue among emergency medicine clinician shift workers. Additional aims include testing whether a theory-based behavioral intervention, delivered by SMS, changes any one of eight factors that are hypothesized to impact ‘alertness behaviors’. These factors are based on the Integrative Model of Behavioral Prediction and include: 1) attitudes, 2) perceived norms, 3) self-efficacy, 4) knowledge, 5) salience, 6) habits, 7) environmental constraints, and 8) behavioral intent. We hypothesize that performance (study subject compliance) with text message assessments will be high and that our SMS behavioral intervention distributed via text messaging will show a reduction in perceived fatigue at the end of shiftwork. We hypothesize that our intervention will impact one or more of the eight factors predictive of alertness behaviors. Study findings will provide important preliminary data to address key questions related to the feasibility and performance of an SMS platform to address sleep and fatigue among shift workers.

## Methods/Design

### Trial design

**Figure 1:**
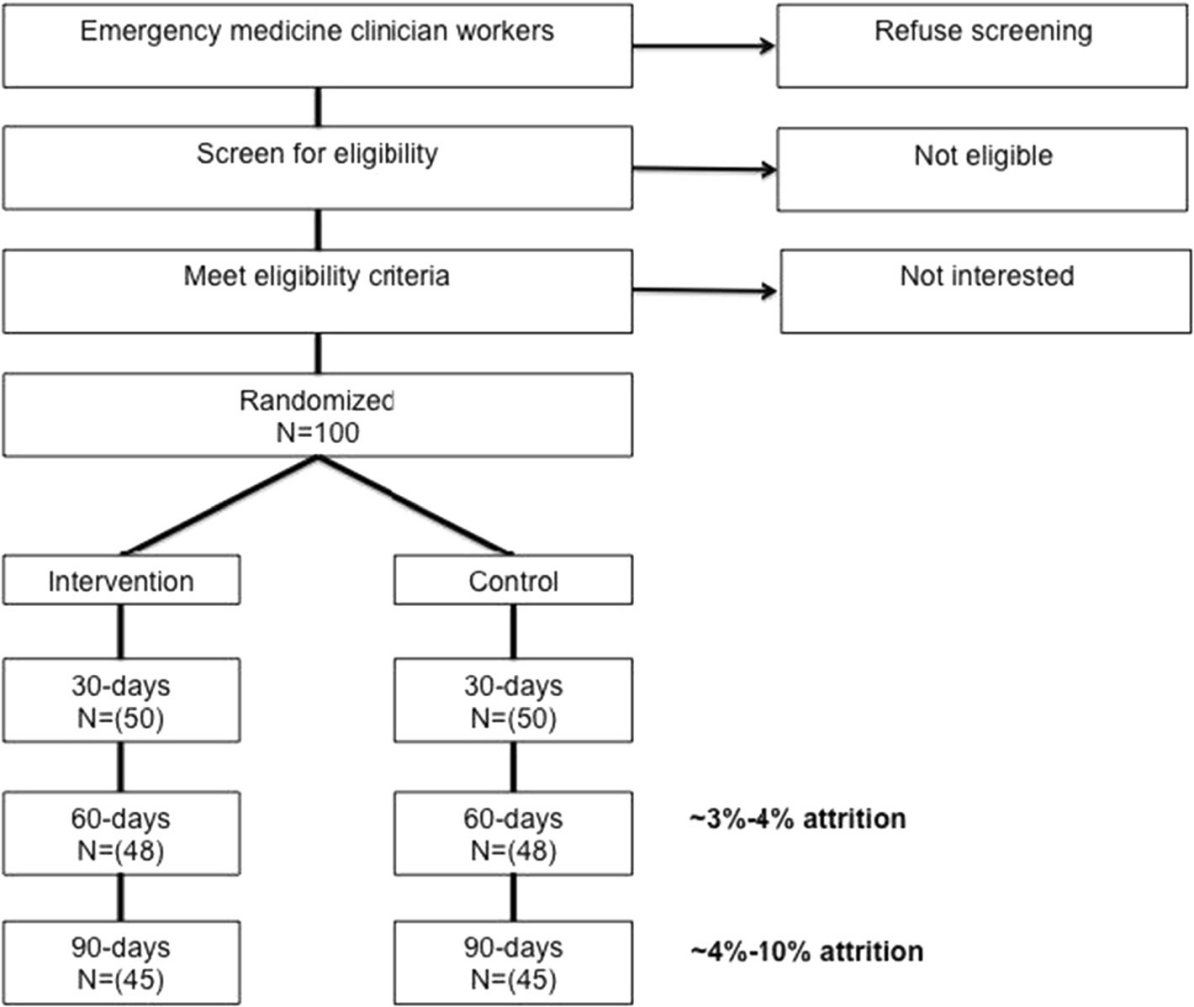
Flow chart of the SleepTrackTXT pilot trial.

The SleepTrackTXT study is a single center, two-arm, parallel, single blind, randomized controlled trial. We use a study webpage as our main means of recruitment. Eligible participants contact the study team to request enrollment and undergo screening. We will randomize 100 emergency workers to one of two groups (N = 50 each). The intervention group will receive 90 days of automated SMS (text messaging) dialogue via a computer. Messages to the subjects will include questions on perceived sleepiness, fatigue, difficulty with concentration, work-related injury at the start, during, and end of scheduled shifts. If subjects in the intervention arm report a high level of sleepiness or fatigue at the beginning of or during their shift the computer recommends one of four evidence-based strategies for improving alertness and reducing sleepiness or fatigue during their shiftwork. The control group subjects will participate in a 90-day SMS dialogue with the computer. In this group, the computer will ask at the start, during, and end of shifts about sleepiness, fatigue, difficulty with concentration, and work-related injury, but the computer will make no recommendations to improve alertness and reduce sleepiness or fatigue. The details of this study design are described below in accordance with the CONSORT statement. Figure 1 provides a visual illustration and flow chart of the SleepTrackTXT trial. This study was approved by the University of Pittsburgh Institutional Review Board and is registered at clinicaltrials.gov (registration number: NCT02063737). All subjects are required to give informed consent.

### Setting

We will begin recruitment using email lists of emergency physicians, emergency nurses, and other emergency technicians working or training at one of three emergency departments (EDs) affiliated with a large academic medical center, the University of Pittsburgh Medical Center in Pittsburgh, Pennsylvania. The recruitment webpage is visible by any emergency worker in the United States. For those outside the academic health system, we anticipate a diverse sample from across the United States will be exposed to study recruitment via our study webpage. The total number of emergency workers expected to have an interest in participating in the study is unknown.

### Recruitment protocol

After an emergency worker views the website an appointment for telephone screening and enrollment is made via email by the emergency worker. If eligible, subjects complete the following steps: 1) text the words ‘Sleep Track TXT’ to the study telephone number to register his or her cell or smartphone with the SMS system; 2) visit the study portal webpage and watch the informed consent video and make a selection to voluntarily participate or not (this is at time of enrollment); 3) if the subject agrees to participate, he/she will create a unique login and password; 3) the subject will then login and answer baseline survey questions that capture demographics and patterns of sleep, fatigue, and shiftwork; 4) the subject will then use a calendar tool to register his or her shift schedule for the next 90 days. Subjects can use the calendar tool to add or edit shifts at any time during the study. Consenting subjects will answer baseline survey questions and provide the contact information of at least one contact if needed for follow-up. This study has been approved by the University of Pittsburgh Institutional Review Board (IRB number: PRO13120428).

### Participants

Inclusion criteria are: 1) 18 years of age and older, 2) working in the emergency care setting as an emergency physician, emergency nurse, emergency medical technician or paramedic, 3) currently working shifts as part of their employment in the emergency care setting, 4) have an SMS-enabled cellphone or smartphone, and 5) are willing to take part in a research study that requires the sending and receiving of multiple text messages at the start, during, and end of shiftwork.

### Randomization

Subject randomization is done using an adaptive algorithm, which will assign participants with a 1:1 allocation, restricting the imbalance to no more than four between the groups. The random number generator and algorithm is embedded in the structured query language (SQL) software program that supports the text message-based intervention. Randomization happens during their first attempt to log into a unique and password protected web-based account connected to the SQL program and complete the informed consent procedures.

### Intervention: text message assessments with recommendations

**Figure 2:**
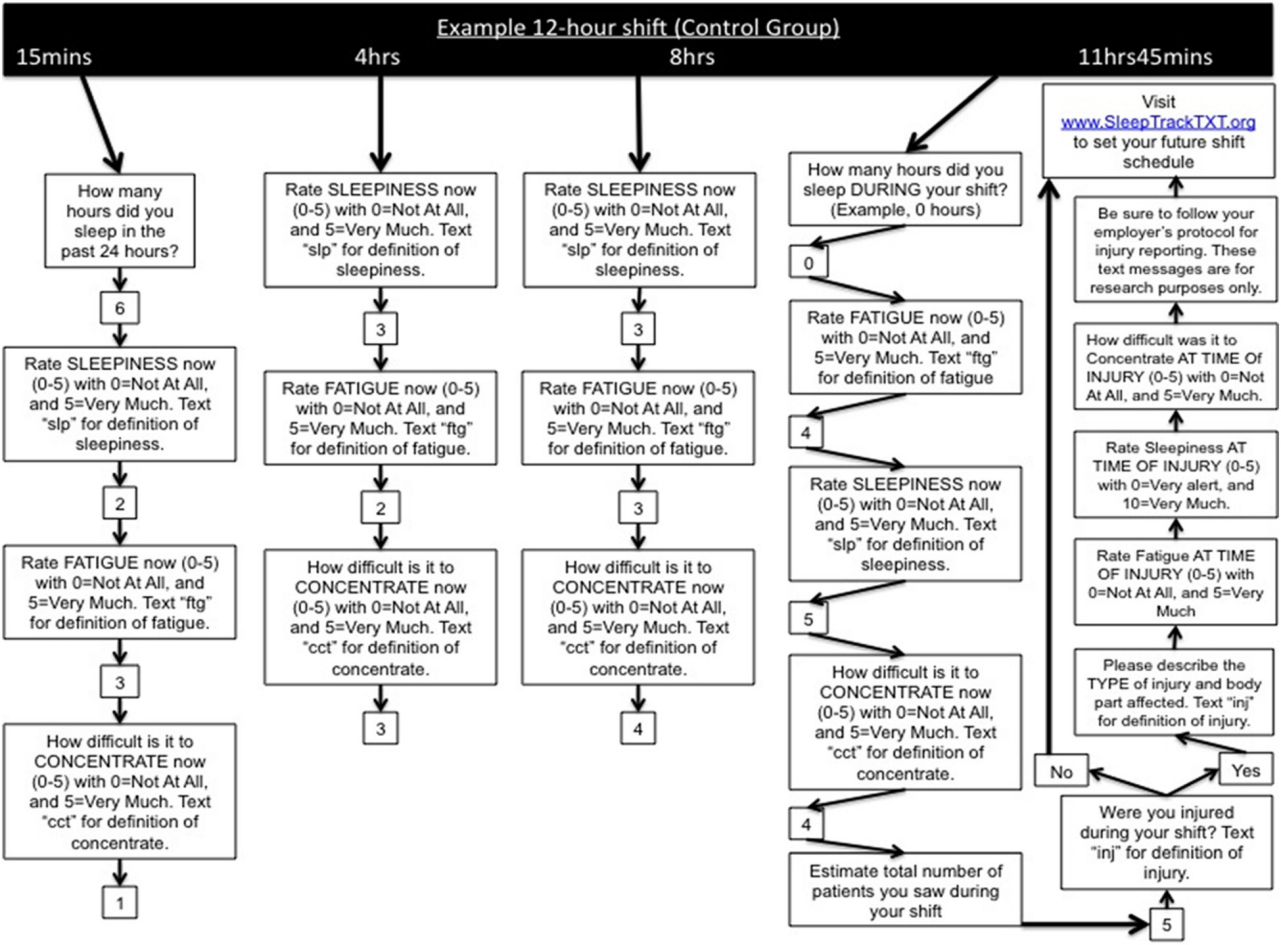
Flow chart of SMS messages for all study subjects.

**Figure 3:**
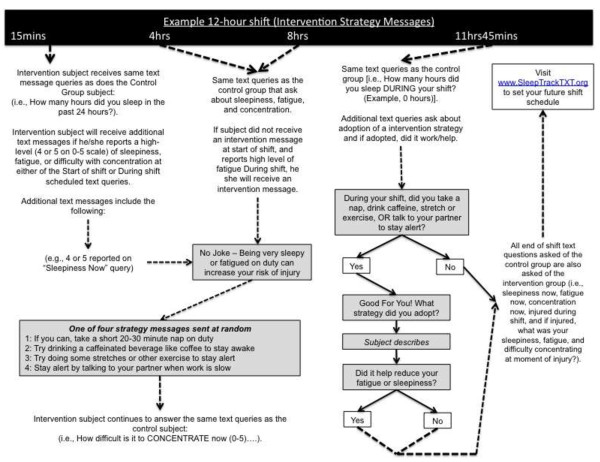
Flow chart of SMS messages for the intervention group.

The intervention group text message recommendations to reduce of sleepiness or fatigue target the following behaviors: 1) naps and rest time [5-10], 2) consumption of caffeine [11], 3) physical exercise [12,13], and 4) use of talking and conversation with partners on duty as a form of mental exercise to stay alert. Our intervention seeks to impact one or more predictors of ‘alertness behavior’ during shiftwork. We operationalize the predictors of alertness behavior and potentially modifiable factors using the Integrated Model of Behavior Change [34]. The model combines components and variables thought to be influential in understanding and predicting behavior [34]. Other models include the Health Belief Model, Social Cognitive Theory, and Theory of Reasoned Action [34]. The key components and variables of these models include: 1) an individual’s attitude, 2) his or her perceived norms, and 3) his or her self-efficacy with respect to performing one or more behaviors. Our intervention consists of four motivating messages or strategies sent to a study subject if he or she reports a high level of sleepiness or fatigue at the start or during their scheduled shift. Upon reporting a high level of sleepiness or fatigue the study subject is sent the following opening text message linking sleepiness or fatigue to bad outcomes: *‘*No joke *-* Being very sleepy or fatigued on duty can increase your risk of injury’. This message is then followed by one of four randomly selected strategies for reducing feelings of sleepiness or fatigue and improving alertness during shiftwork: 1) If you can, take a short 20-30 minute nap on duty; 2) Try drinking a caffeinated beverage like coffee to stay awake; 3) Try doing some stretches or other exercise to stay alert; 4) Stay alert by talking to your partner when work is slow. We developed the intervention strategy messages based on prior research showing a positive impact on perceived sleepiness, fatigue, or alertness [5-13]. The SMS-enabled dialog and strategy messages were developed by study investigators and are hypothesized to have an impact on the behavioral determinants of an emergency worker’s intention to maintain alertness and vigilance during shiftwork. Figures 2 and 3 provide an illustration and flow chart of SMS messages for all subjects (Figure 2) and intervention content (Figure 3).

### Control: text messaging assessment only

Individuals in the control group will not receive any recommendations for improving alertness and reduction in intra-shift sleepiness or fatigue (Figure 2). Control subjects will receive text messages for assessment only at the beginning, during, and end of shifts.

### Remuneration

All participants will receive the following incentives: a coffee mug and $10 gift card at completion of enrollment, a $10 gift card at completion of 30 days, and again at completion of 60 and 90 days, and entry into a drawing for one of five tablet computers for completion of all components of the study. We believe this plan will incentivize adherence without creating unrealistic expectations for compliance in further studies.

### Measures

#### Participant level measures

After completion of the informed consent process we will collect demographic data on age, sex, alcohol consumption, smoking status, perceived general health, employment status, and eight other demographic factors. Other baseline measures will include sleep quality, fatigue, attitudes about shift scheduling, and perceptions of work-related fatigue and recovery (see Table 1). Sleep quality will be measured with the widely used and reliable Pittsburgh Sleep Quality Index (PSQI) [35]. A PSQI score of 6 or more on a 21-point scale indicates poor sleep quality [35]. Mental and physical fatigue at work will be measured using the Chalder Fatigue Questionnaire (CFQ) [36]. The CFQ is an 11-item scale of mental and physical fatigue that has been used in multiple emergency worker populations and shown by psychometric testing to be reliable and valid [15,16]. A CFQ score exceeding one of two scoring benchmarks can indicate severe mental and physical fatigue. Attitudes about shift scheduling will be measured with the Schedule Attitudes Survey (SAS) [37]. The SAS is a 21-item scale that measures a worker’s attitudes about his or her shift schedule. The tool’s intended use was for schedule modification and design by employers. The SAS has been used in diverse populations, including EMS workers, and is shown to have positive psychometric properties of reliability and construct validity [20]. Higher scores on the SAS are interpreted as representing greater dissatisfaction towards one’s current shift schedule.

**Table 1:** Schedule of measurements for the study in SleepTrackTXT pilot trial

**Measure**	**At baseline enrollment**	**Beginning of every scheduled shift**	**Every 4 hrs during scheduled shifts**	**End of every scheduled shift**	**Subject reports an injury (at end of shift, or at any time)**	**At 90-days end of study**
Demographic survey	X					
Pittsburgh Sleep Quality Index (PSQI)	X					X
Chalder Fatigue Questionnaire (CFQ)	X					X
Schedule Attitudes Survey (SAS)	X					X
Occupational Fatigue, Exhaustion, Recovery Scale (OFER)	X					X
Sleep, Fatigue, Alertness Behavior Survey (SFAB)	X					X
Hours of sleep in past 24 hours		X				
Rate sleepiness now		X	X	X		
Rate fatigue now		X	X	X		
How difficult is it to concentrate now		X	X	X		
Estimate total number of patients you saw during your shift				X		
Were you injured during your shift?				X	X	
Describe the type of injury and body part affected*				X*	X*	
Rate sleepiness at time of injury*					X*	
Rate fatigue at time of injury*					X*	
How difficult was it to concentrate at time of injury*					X*	
**Intervention group only**^**a**^						
During your shift, did you take a nap, drink caffeine, stretch or exercise, or talk to your partner to stay alert?				X		
What strategy did you adopt?				X		
Did it help reduce your fatigue or sleepiness?				X		

*if subject reports an injury.

^a^These measures are administered to the intervention group when a subject reports a high level of sleepiness, fatigue, or difficulty concentrating at the beginning or during shift assessments.

Work-related fatigue and recovery will be measured with the Occupational Fatigue Exhaustion/Recovery Scale (OFER) [38]. The OFER is a reliable and valid 15-item scale that measures acute fatigue, chronic fatigue, and inter-shift recovery. Higher OFER scores ( ≥80) indicate higher fatigue and greater ability to recover between shifts. At baseline and at 90-days follow-up we will measure a participant’s behavior and intention to engage in behaviors that improve alertness with the Sleep, Fatigue, & Alertness Behavior Survey (SFAB). We developed the SFAB based on the Integrated Model of Behavioral Prediction [34]. The SFAB is comprised of 83 candidate items intended to measure eight factors and domains considered to be predictive of behavioral intent to improve alertness during shiftwork. These eight domains include: 1) attitudes about improving or maintaining alertness on duty; 2) perceived norms regarding what the person or others (peers) think or feel about being sleepy or fatigued while at work; 3) self-efficacy regarding improving or maintaining alertness while on duty; 4) knowledge of behaviors for improving or maintaining alertness on duty; 5) salience (perceived importance) of specific alertness behaviors; 6) habits linked to sleepiness, fatigue, and alertness; 7) environmental constraints to performing specific alertness promoting behaviors; and 8) intention to perform one or more behaviors. We will administer the SFAB at baseline and at 90-days follow-up to categorize individuals into one of four behavioral categories and assess the following changes in behavior: 1) intention to reduce fatigue and sleepiness on duty and reports consistent use of alertness-promoting behaviors; 2) intention to reduce fatigue and sleepiness on duty and reports inconsistent or no use of alertness-promoting behaviors; 3) no intention to reduce fatigue or sleepiness on duty and reports consistent use of alertness-promoting behaviors; and 4) no intention to reduce fatigue or sleepiness on duty and reports inconsistent or no use of alertness-promoting behavior.

#### Shift level measures and primary outcome

Our performance outcome of interest is subject compliance with text message assessments. Our primary intervention outcome of interest is self-reported fatigue at the end of scheduled shifts. Single-item measurement is the preferred approach for research that involves real-time data capture and/or ecological momentary assessment (EMA) [39,40]. We based the construction of our single-item outcome measure and the scale for respondents to record their subjective response on prior EMA research of insomnia [41]. We hypothesize that study subjects in the intervention group will report lower levels of fatigue at the end of shift than study subjects in the control group.

#### Follow-up measures

Each study subject will complete the following survey assessment tools at 90 days: the PSQI, CFQ, SAS, OFER, and SFAB.

#### Sample size

In planning the study, several unknowns prevented a well-informed sample size analysis including the number of shifts each participant would contribute and the amount of correlation in fatigue between multiple shifts within the same subject. We assumed that compliance would be high for all study subjects, with over 80% of text message assessments answered [42]. Due to the pilot nature of the study, we conservatively assumed a minimum of one observation per person and then determined the number we would need to see a large effect size for the ‘proof-of-concept’ outcome. Based on our previous cross-sectional data [15,16] we assumed that 50% of EMS workers would report fatigue at the beginning of or during shift (at risk population). If N = 25 per study arm, we will have 80% power to detect a large effect size of 0.78 difference in the mean end of shift fatigue score between control and intervention groups (alpha = 0.05), using a two-sample Mann-Whitney U test power analysis (PASS software, NCSS Statistical Software, Kaysville, Utah, United States) assuming the actual distribution is logistic. Given the 50% expected rate of fatigue at baseline, we will recruit and randomize 100 participants (50 per group) to obtain the ‘at risk’ number needed within each arm.

#### Statistical analysis

We will describe overall number of shifts per provider and the mobile text participation for all study subjects. For example, we will quantify compliance with our text message queries and the number that were lost to attrition. We will use linear mixed-model analyses to examine the effectiveness of the intervention on reducing reported fatigue at the end of shift among all participants and among only those reporting fatigue at baseline. We will construct a model where the outcome is the reported fatigue at the end of each shift, a fixed effect for experimental group, a fixed effect of time in study, and a random effect for subjects to account for multiple shifts within each person. We will examine the estimated change in fatigue score per shift in the intervention group compared to the control group. Post-estimation testing will be used to describe the correlation in fatigue scores within the subjects across shifts. The same methodology will be used to analyze end of shift sleepiness and difficulty in concentrating.

The eight scale components of the SFAB survey are measured at baseline and at the end of the study. Likert responses will be scored as continuous measures and weighted from 0 to 100, with higher scores indicating more positive scores. We will test for changes over time and between-group differences in these scales using one sample and two sample t-tests, assuming the underlying distribution of the outcomes are normal. If the data are highly skewed we will use non-parametric tests including the sign rank test and Mann-Whitney U test. All statistical testing will be two-sided with alpha set at <0.05 using STATA version 10 SE (StataCorp LLP, College Station, Texas, United States) and SAS version 9.2 (SAS Institute Inc., Cary, North Carolina, United States).

## Discussion

The SleepTrackTXT pilot trial could provide evidence of compliance and effectiveness that would support rapid, widespread expansion in one of two forms: 1) a stand-alone program in the form of a tailored/individualized worker sleep monitoring and fatigue reduction support service; or 2) an add-on to a multicomponent fatigue risk management program led and maintained by employers or by safety and risk management services.

Our study design has several strengths. We will characterize the performance of an SMS tool for the purpose of assessing sleep and fatigue in real-time among a highly vulnerable shift worker population. We will provide a detailed picture of variability in sleepiness and fatigue over the course of shiftwork and expose moments of greatest need for intervention. This is significant given that the existing literature, especially involving the prehospital emergency medical services clinicians, is largely limited to cross-sectional study designs [43]. These cross-sectional data may be misrepresented as typical of an emergency clinician’s state of sleepiness or fatigue over the entirety of a shift, no matter shift length or volume of work. Existing interventions or agency level policies based on cross-sectional data may be misinformed and ineffective. Capturing variability in a clinician’s condition over the course of an 8, 12, 16, 24-hour or longer shift schedule exposes points in time more proximal to when a subject may be at greatest risk of a negative outcome and the opportunity when an intervention may realize its greatest impact and benefit.

Second, we have a unique opportunity to examine the impact of our intervention on emergency clinician behavior and behavioral intent. In the Methods/Design section, we described the theory-driven SFAB survey tool and how its component parts measure multiple factors believed (based on extensive theoretical literature [34]) to predict intention to perform a behavior that may improve alertness while on duty. All participants will complete a baseline assessment that measures numerous aspects of their sleep and shiftwork patterns and factors that are linked to behavioral intention, including attitudes, perceived norms, self-efficacy, knowledge, salience, environmental constraints, habits, and other factors linked to behavior. By collecting these data at baseline and again at the end of the study period, we have the opportunity to identify subgroups of emergency clinicians that achieve the greatest benefit based on our current intervention, and to identify subgroups that require a special intervention tailored to their unique classification according to the SFAB.

Our study offers a novel, practical, and scalable solution to a controversial issue, the fatigued emergency worker and threat of extended shifts. Present day solutions to worker fatigue adopted by many administrators involve changing shiftwork practice patterns, such as reducing or eliminating extended shifts [44,45]. Reducing shift length is contentious and may not have the impact desired. There is limited data from emergency worker groups that show a short- or longterm positive benefit to reducing shift length [20]. Many in the EMS setting already work multiple jobs, with more than 80% in some locations work at more than one EMS organization [14]. A change in shift length policy industry-wide may exacerbate the problem by causing more EMS workers to seek additional employment.

These data will offer preliminary answers to common questions such as: how severe are sleepiness and fatigue at the end of a 24 hour shift versus a shift of shorter length? The main methodological challenge will be subject compliance with text message assessments occurring during the shift period at intervals of every four hours. Many clinicians will be busy with patient care or other activities and find it difficult to respond. Study subjects working longer shifts (for example a 24 hour shift) will be set to receive many more text messages than subjects working shifts of a shorter length. Text messages may be perceived as repetitive and there is limited human contact with the study team. The ideal number of text messages per shift or per day is unknown. One study reports study subjects’ perceived ideal total number of daily text messages is 5.5 (range 1 to 20) [46]. Attrition over time is another challenge. Recent studies involving SMS-based assessments and interventions report attrition or withdrawal rates of 2.4 to 3.2% [46,47]. Studies of sensitive subjects such as sexually transmitted disease report lower longterm adherence and compliance and higher attrition [48]. Sleep and fatigue are highly visible topics within the EMS industry that are controversial when the topic of shift length restriction is discussed [43]. This is the first study of its kind and it is unclear if the noted controversy related to shift length restriction would impact recruitment or attrition. We have included a modest incentive program to address the threat of attrition. We have included an end of shift question about participant workload, which will be used in the analysis as a stratification variable for different levels of compliance with scheduled queries during shiftwork.

## Trial status

At time of manuscript acceptance, enrollment was closed and follow-up data were being collected.

## Abbreviations

CFQ: Chalder fatigue questionnaire; ED: Emergency department; EMA: Ecological momentary assessment; EMS: Emergency medical services; mHealth: Mobile health; OFER: Occupational fatigue, exhaustion/recovery scale; PSQI: Pittsburgh sleep quality index; SAS: Schedule attitudes survey; SFAB: Sleep fatigue & alertness behavior survey; SMS: Short message service; SQL: Structured query language.

## Competing interests

All authors report no competing interests or conflicts of interests. In the past five years, author PDP has received grant support from the National Institutes of Health (NIH), Centers for Disease Control’s National Institute for Occupational Safety and Health (CDC, NIOSH), Federal Emergency Management Association (FEMA), and foundations including the North Central EMS Institute (NCEMSI), Pittsburgh Emergency Medicine Foundation (PEMF), and Jewish Healthcare Foundation (JHF). Authors CWC, CGM, and DJB report grant support from NIH over previous five years. Author DMY reports recent grant support from NIGMS and NHLBI. Author BPS reports grant support from the American College of Emergency Physicians’ Emergency Medicine Foundation (ACEP EMF). The conclusions, views, opinions, and content in this paper should not be interpreted as reflecting the opinions of the NIH, NIGMS, NHLBI, CDC NIOSH, NCEMSI, PEMF, JHF, or the ACEP EMF.

## Authors’ contributions

PDP, BPS, DJB, and MDW conceived the study idea and framework. PDP, CGM, BPS, DJB, MDW, CWC, and DMY participated in development of the study protocol, data collection tool, and study methodology. PDP, CGM, and MDW conceived the planned statistical analysis. All authors participated in development of draft manuscripts and editing of the final manuscript. All authors have read and approve the final manuscript.
